# To What Extent Is Water Responsible for the Maintenance of the Life for Warm-Blooded Organisms?

**DOI:** 10.3390/ijms10052383

**Published:** 2009-05-22

**Authors:** Anatoliy I. Fisenko, Nikolay P. Malomuzh

**Affiliations:** 1 Oncfec, Inc., 625 Evans Avenue, Suite 1108, Toronto, Ontario, M8W 2W5, Canada; 2 Department of Theoretical Physics, Odessa National University, 2 Dvoryaskaja Street, Odessa, 65026, Ukraine; E-Mail: mnp@normaplus.com

**Keywords:** intracellular and extracellular fluids, upper physiological temperature limit, lower physiological pH limit, H-bond network, warm-blooded organisms, translational and rotational thermal motions, entropy, kinematic shear viscosity, normal and heavy water, smeared dynamic phase transition, water-glycerol solutions, isothermic compressibility, cancer tissues, blood, denaturation of proteins, ions, electrolyte solutions, birds

## Abstract

In this work, attention is mainly focused on those properties of water which are essentially changed in the physiological temperature range of warm-blooded organisms. Studying in detail the half-width of the diffusion peak in the quasi-elastic incoherent neutron scattering, the behavior of the entropy and the kinematic shear viscosity, it is shown that the character of the translational and rotational thermal motions in water radically change near *T_H_* ~ 315 K, which can be interpreted as the temperature of the smeared dynamic phase transition. These results for bulk pure water are completed by the analysis of the isothermic compressibility and the NMR-spectra for water-glycerol solutions. It was noted that the non-monotone temperature dependence of the isothermic compressibility (*β_T_*) takes also place for the water-glycerol solutions until the concentration of glycerol does not exceed 30 mol%. At that, the minimum of *β_T_* shifts at left when the concentration increases. All these facts give us some reasons to assume that the properties of the intracellular and extracellular fluids are close to ones for pure water. Namely therefore, we suppose that the upper temperature limit for the life of warm-blooded organisms [*T_D_* = (315 ± 3) K] is tightly connected with the temperature of the dynamic phase transition in water. This supposition is equivalent to the assertion that the denaturation of proteins at *T* ≥ *T_H_* is mainly provoked by the rebuilding of the H-bond network in the intracellular and extracellular fluids, which takes place at *T* ≥ *T_H_*. A question why the heavy water cannot be a matrix for the intracellular and extracellular fluids is considered. The lower physiological pH limit for the life of warm-blooded organisms is discussed.

## 1. Introduction

The surprising properties of water have been the subject of extensive research for many years [1,2]. Usually it retains the non-monotone temperature dependencies of density, isothermic compressibility and thermoconductivity as well as the large values of dielectric permittivity, essentially larger than for the majority of polar liquids. Moreover, special attention is paid to the differences in the behavior of the binary correlation function for water and simple liquids, and the increase of density at the melting point. All these peculiarities are a reflection of the existence of H-bonds in water.

Another kind of the surprising properties of water have been discovered [3–5]. Studying the peculiarities of the diffusion peak in the quasi-elastic incoherent neutron scattering in water and the comparative behavior of the kinematic shear viscosity in water and argon it had been shown that the character of the thermal motion in water is essentially changed at *T_H_* ≈ (42 ± 3) °C. At *T < T_H_*, it is a crystal-like. In the opposite case, when *T > T_H_*, the character of the thermal motion becomes to be argon like, i.e. the oscillations near the temporary equilibrium positions disappear.

Taking into account the closeness of *T_H_* to the upper death temperature *T_D_* for warm-blooded organisms, in [6,7] it was supposed that their death is caused by the change of the character of the thermal motion in the intracellular and extracellular fluids, which we call the body fluids. This circumstance is self-evident since the life activity of proteins strongly depends on processes of the mass-, ions-, and energy exchanges with the surrounding intracellular fluid.

**Figure 1.**
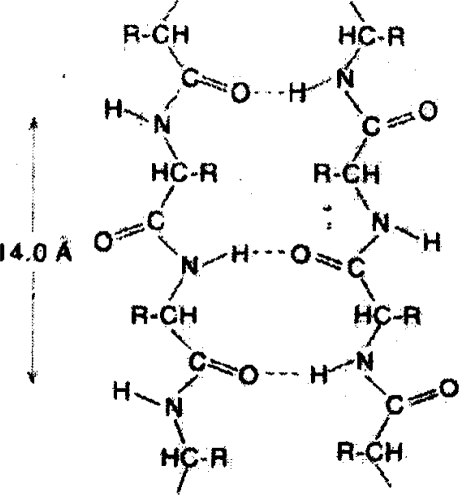
Fragment of a RNA chain in the cytoplasm of a cell (from [8]).

As seen in Figure 1, the interaction between links of a protein macromolecule and their interaction with the surroundings is mainly put into effect by bridges of the type *O–H···N*, whereas in water the elementary H-bond is *O–H···O*. However, the difference between them is not essential [9].

In bulk water, as well as in the intracellular fluid far away from the protein surface, H-bonds form the bulk network, while its geometry close to the protein surface is confined. However, one can hope that the local structure of the H-bond network will be insensitive to the geometrical restrictions.

Here it is necessary to pay attention to the following important fact: the properties of the H-bond network in bulk water change essentially with temperature and pressure, and they influence the character of the thermal motion of molecules in water and water solutions. A molecule can oscillate only near some temporary equilibrium position if the average number *n_H_* of H-bonds per molecule is equal to three or greater. In this case, the thermal motion of molecules has crystal-like character. The shift of a molecule to another position is possible if one or two H-bonds break. If *n_H_* is reduced to two or becomes smaller, which occurs at *T > T_H_*, the thermal motion in water recalls that in simple liquids, in particular argon. Therefore, the study of properties of the H-bond network in water and water solutions, especially in the temperature interval of the life for warm-blooded organisms, is a very important problem. In general, the existence of H-bond network in water manifests itself in the different peculiarities of its behavior.

The properties of the intracellular and extracellular fluids, as it seems at first sight, should be essentially differing from those for bulk pure water. In this situation, the analysis of properties of simpler model systems can help to understand the role of different factors. In this paper, we will examine the properties of water-glycerol solutions. They were the object of the detailed study reported in [10,11]. In particular, the temperature dependences of density and isothermic compressibility in them at different concentrations were investigated. It was shown that for a large enough mass concentrations of glycerol, the solution demonstrates properties genetically connected with properties of water. Due to this, one can suppose that a similar situation also takes place for the intracellular and extracellular fluids.

In this paper, we present different evidence for the existence of the dynamic phase transition at *T_H_* *~ T_D_* in bulk water and the extracellular and intracellular fluids. Studying the non-equilibrium properties such as the spectrum of the quasi-elastic incoherent neutron scattering and the kinematic shear viscosity as well as the behavior of the entropy diameter, we will obtain several independent estimates for *T_H_*. Based on the analysis of the temperature dependencies for the specific volume and the heat of evaporation per molecule, we will reach important conclusions about the properties of the H-bond network in water and the character of the rotational motion of water molecules. The properties of the water-glycerol solutions, obtained from the study of the elastic reaction of a system and with help of NMR, will allow us to conclude that the behavior of the intracellular and extracellular fluids is similar to that for pure water in many aspects. To better understand the role of normal water as the matrix for intracellular and extracellular fluids the comparison of some important properties of normal and heavy water is performed. The paper ends with a detailed discussion of the results obtained.

## 2. Dynamic Phase Transition According to the Quasi-elastic Neutron Scattering Data

This Section is devoted to the consideration of the thermal motion in water as the function of temperature. We start from situation taking place near the melting point as well as in supercooled states, where the local structure of water is close to the regular structure in the hexagonal ice [12]. The similarity of the thermal motion in liquid water and hexagonal ice is also expected.

**Figure 2.**
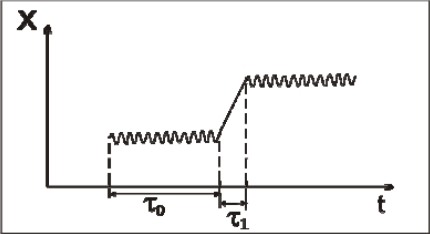
Schematic picture for the crystal-like character of the thermal motion in water.

Let *τ*_0_ be the characteristic time for small oscillations of a molecule near its temporary equilibrium position. Often, this time is called as the residence time [13]. The characteristic time *τ*_1_, during which a molecule displaces from an initial vibration state to another one, will be called as the transition time. The character of small oscillations in supercooled water is close to that in the hexagonal ice, where each molecule is connected with its nearest neighbors by four H-bonds. However, the duration of *τ*_0_ in them is different: *τ*_0_ = ∞ in the hexagonal ice and it takes a finite value for supercooled and normal water. One can say that the thermal motion in water has a crystal-like character if *τ*_0_ ≫ *τ*_1_. This situation is illustrated in Figure 2.

An increase in temperature leads to the diminution of *n_H_*. However, while *n_H_* > 2, H-bonds remain ordered in the three-dimensional H-bond network.

In accordance with its physical meaning, the transition time *τ*_1_ should be identified with the characteristic time of soft collisions between molecules: *τ_s_* *~ a/υ_T_*, where *υ_T_* is the average value of the thermal velocity of a molecule. This value is diminishes only slightly when temperature increases. In contrast, the residence time varies considerably more. It decreases when temperature increases and its value tends to the transition time *τ*_1_.

The temperature *T_n_*, defined as the solution of the equation:

(1) $$\tau_{0}(T_{n})=\tau_{1}(T_{n}),$$

which specifies the upper temperature limit for the applicability of the crystal-like representations. In other words, it can be interpreted as the temperature of the dynamic phase transition in water: from the crystal-like motion of molecules to the argon-like one.

For temperatures *T* > *T_n_*, the crystal-like representations for the thermal motion in water become no longer applicable. In this temperature region, the character of the thermal motion is similar to that in simple liquids, where molecules interact by spherically symmetrical interparticle potentials.

The value *τ*_0_, as well as its temperature dependence, can be reliably determined with the help of experimental data on the quasi-elastic incoherent scattering of cold neutrons.

In general, the spectrum of the incoherent neutron scattering consists of the relatively narrow diffusion peak and the wide constituent usually considered as a background. The description of the diffusion peak in the framework of the crystal-like representations, given in [14], is not quite correct, since the shift of a molecule during time *τ*_1_ is not described by the diffusion law. Therefore, in Supplement 1 we briefly modify the derivation for the half-width of the diffusion peak. We will also take into account that the diffusion approximation is applicable only for the wave vectors $\vec{k}$ satisfying the inequality: | $\vec{k}$ | *a* ≪ 1, where *a* is the interparticle spacing (see details in [3–5]).

In accordance with Equation (31), the half-width *γ_D_* ( ${\vec{k}}^{2}$) is given by the expansion:

(2) $$\gamma_{D}({\vec{k}}^{2})\approx D_{s}{\vec{k}}^{2}-\tau_{0}D_{s}^{{\left(1\right)}^{2}}{\vec{k}}^{4}+\tau_{0}^{2}D_{s}^{{\left(1\right)}^{3}}{\vec{k}}^{6}+\ldots ,$$

where $D_{s}^{\left(1\right)}$ is the one-particle contribution to the full self-diffusion coefficient *D_s_*, *D_c_* is its collective part, $D_{s}=D_{s}^{\left(1\right)}+D_{c}$, ${\vec{k}}^{2}$ is the square of the transfer wave vector.

**Figure 3.**
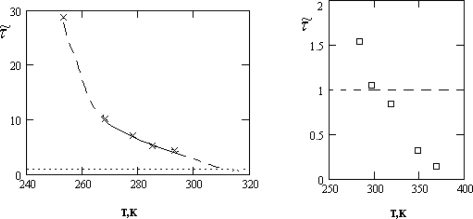
The temperature dependence of the ratio *τ͂ =* *τ*_0_*/τ*_1_: crosses correspond to the experimental data [15], squares – to [16] (the value *τ*_0_ is determined with the help of Equation (2)).

Fitting the diffusion peak of the incoherent neutron scattering with the help of Equation (2), we can determine *D_s_* and $D_{s}^{\left(1\right)}$ as well as the residence time *τ*_0_. The temperature dependence of the ratio. *τ͂ =* *τ*_0_*/τ*_1_ is presented in Figure 3.

As follows from Figure 3, the inequality *τ*_0_ ≫ *τ_s_*, required for the applicability of the crystal-like representations, is reliably satisfied only in the supercooled region and for the normal states of water near the crystallization point, *T* > *T_n_* = 315*K*.

Below, we will show that the characteristic changes of the thermal motion at temperatures close to *T_n_* manifest themselves also in other properties of water. This circumstance gives us reason to assert that the character of the thermal motion in water at *T_H_* = (315±3)*K* undergoes a specific transformation, which will be qualified by us as the dynamic phase transition.

It is noteworthy that the rapid increase of the residence time τ_0_ as the temperature decreases can be naturally interpreted in the framework of cluster representations, especially characteristic for supercooled states. Indeed, it had been shown in [17] that the relative volume occupied by the crystal-like clusters increases from the value *ϕ* = 0.11 at the melting temperature *T_m_* = 273*K* up to *ϕ* = 0.41 at *T* = 243*K*. For these states of water τ_0_ can be actually identified as the lifetime of the crystal-like clusters. Their average size changes more slowly and remains close to 10 Å. Note that among clusters, the leading role belongs to the hexagonal rings, which are the building elements for the ordinary (hexagonal) ice. Probably, *φ* → 0 when *T → T_H_*. As we will see below, the clusterization essentially influences the mobility of ions. This circumstance is also important for the thermal motion of ions inside cells.

The crystal-like picture of the thermal motion in water near the melting point is also supported by the results of computer simulations presented in [18]. There it was shown that for *T* < 284 K the increment of the mean square displacement $<\Delta {\vec{r}}^{2}(t)>$ of a molecule is close to zero in the time interval 10^−13^ *s* ≤ *t* ≤ *τ*_0_ where *τ*_0_ > 10^−12^ s, starting from *T* ≤ *T_m_*. Unfortunately, higher temperatures were not considered in [18]. Important information about the dynamic phase transition in water at *T* ~ *T_H_* can be obtained from the temperature dependence of the kinematic shear viscosity *ν*(*T*).

## 3. The Determination of *T_H_* from the Kinematic Shear Viscosity of Water

The kinematic shear viscosity of liquids is one of their main transport coefficients. It is formed by different constituents of the thermal motion of molecules in liquids, in the first place, by the translational and rotational degrees of freedom. For water, the considerable influence on their manifestation is produced by H-bonds. Thus, if a molecule is connected with its nearest neighbors by three or four H-bonds, it can only oscillate near some temporary equilibrium position.

**Figure 4.**
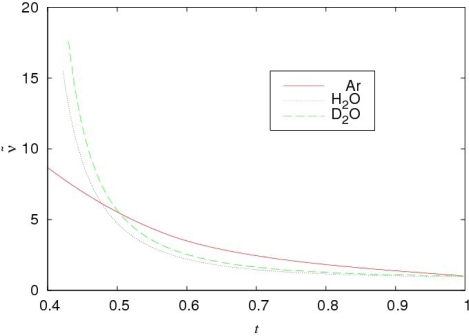
Temperature dependencies of the normalized kinematic shear viscosities for argon and the normal and heavy water. The experimental data are taken from [23–25].

For separating contributions of different physical nature, let us compare the behavior of the normalized shear viscosities for water and argon in the manner of the principle of corresponding states [19–21]. The normalized values of the kinematic shear viscosities are determined as:

$${\tilde{v}}^{\left(i\right)}(t)=\frac{v^{\left(i\right)}(t)}{v_{R}^{\left(i\right)}}\;\;\text{and}\;\tilde{v}(t)=\frac{v^{\left(Ar\right)}(t)}{v_{R}^{\left(Ar\right)}},$$

where $\text{t}=\text{T}/T_{c}^{\left(i\right)}$, $T_{c}^{\left(i\right)}$ are the critical temperatures for normal and heavy water, and argon, $v_{R}^{\left(i\right)}$ are their regularized values at $T_{c}^{\left(i\right)}$ (see details in [21,22]) and *i* = *H*_2_*O*, *D*_2_*O*. The temperature dependencies of *ν͂*^(*H*_2_*O*)^ (*t*), *ν͂*^(*D*_2_*O*)^ (*t*) and *ν͂*(*t*) are presented in Figure 4.

The points of intersection *t_ν_* for the curves *ν͂*^(^*^i^*^)^ (*t*), *i = H*_2_*O*, *D*_2_*O*, and *ν͂*(*t*) are the characteristic temperatures for water. They separate two temperature intervals, in which the behaviors of the kinematic shear viscosities are determined by essentially different mechanisms. From the equation:

(3) $${\tilde{v}}^{\left(i\right)}(t_{v})={\tilde{v}}_{ext}(t_{v}),$$

where *ν͂_ext_* (*t*) denotes the extrapolated values of the kinematic shear viscosity of argon in its supercooled region, it follows that:

$$\begin{array}{l} t_{v}(H_{2}O)=0.478\Rightarrow T_{v}(H_{2}O)=309K,\\ t_{v}(D_{2}O)=0.503\Rightarrow T_{v}(D_{2}O)=324.1K. \end{array}$$

As we see, the characteristic temperature *T*_v_(H_2_O) = 309 K for normal water is very close to *T*_H_. Besides, *T*_v_ separates two temperatures ranges with the different character of the temperature dependence of the kinematic shear viscosity. Therefore, it is natural to assume that *T*_v_ and *T_n_* correspond to the same physical phenomenon, which takes place at *T*_H_ ≈ (315 ± 3) K.

In [21,22] it was shown that the kinematic shear viscosity of normal water *ν͂*^(^*^H^*^_2_^*^O^*^)^ (*t*) for the whole temperature interval of liquid water, including supercooled states and the critical point, can be approximated by the formula:

(4) $${\tilde{v}}^{\left(H_{2}O\right)}(t)=(1-\xi )\tilde{v}(t)+{\tilde{v}}_{0}^{\left(H\right)}\exp(\varepsilon /t)-\kappa n_{H}(t),\;\;n_{H}(t)=4(1-\gamma t)$$

where *ζ =* −4*κ*(1 – *γ*). The first term in Equation (4) describes the argon-like contribution, the third one – the contribution stimulated by H-bonds. It has the negative sign since the formation of H-bonds diminishes the translational and rotational motilities of molecules. The second term is connected with the rupture of H-bonds, limiting the motion of molecules. It is dominating in the supercooled region, where the average number of H-bonds per molecules *n_H_* (*t*) is noticeably greater than two. The constants *κ* and *γ*, determined by fitting the experimental data [21,22] with the help of Equation (4), are equal to: *κ* ≈ 1.07 and *γ* ≈ 0.88. It is very essential to note that the estimate:

$$n_{H}(t)=4(1-0.88t)+\ldots ,$$

following from the analysis of the kinematic shear viscosity, is in quite satisfactory agreement with one *n_H_* (*t*) = 4(1 – 0.85*t*) +..., obtained in [26,27] from the analysis of the fraction volume (see also the Section 8).

The behavior of *ν͂*^(^*^D^*^_2_^*^O^*^)^ (*t*) on its coexistence curve has the analogous character that is evident from Figure 4.

## 4. Peculiarities of the Rotational Motion of Molecules in Water

**Figure 5.**
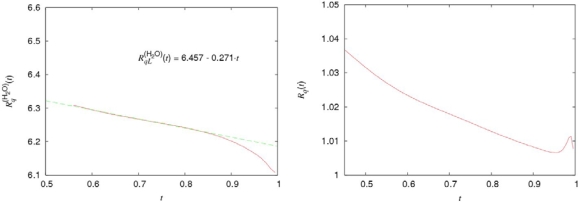
The temperature dependencies of the ratio $R_{q}^{\left(H_{2}O\right)}(t)$ and *R*_q_(*t*) = *q* ^(D_2_O)^ (t)/*q*^(H_2_O)^ (*t*), according to the experimental data [23–25].

Let us complete the analysis of the water density on the coexistence curve by the consideration of peculiarities in the behavior of the water entropy. The combination:

$$q^{\left(w\right)}(t)=k_{B}T(S_{\upsilon }^{\left(w\right)}(t)-S_{l}^{\left(w\right)}(t)),$$

where $S_{v}^{\left(w\right)}(t)$ and $S_{l}^{\left(w\right)}(t)$ are the values of entropy per molecule in the vapor and liquid phases, determines the heat of evaporation of water (per molecule). From Figure 5 (a and b) it follows that for both normal and heavy water the ratio $R_{q}^{\left(i\right)}(t)=q^{\left(i\right)}(t)/q^{\left(Ar\right)}(t)$, (*i* = *H*_2_*O*, *D*_2_*O*) is approximated by the linear functions:

(5) $$R_{q}^{\left(i\right)}(t)=p_{q}^{\left(i\right)}+r_{H}^{\left(i\right)}n_{H}^{\left(i\right)}(t),\;n_{H}^{\left(i\right)}(t)=4(1-\lambda_{i}t),\;\;\lambda_{i}\leq 1.$$

everywhere except the narrow vicinity of the critical point. Here $n_{H}^{\left(i\right)}(t)$ denotes the average number of H-bonds per molecule. The possibility of such a representation for the H-bond contributions to the thermodynamic quantities is discussed in details in [5,26,27] and the Section 7. The numerical values of the coefficients for the normal water are:

(6) $$p_{q}^{\left(H_{2}O\right)}=6.134,\;\;r_{H}^{\left(H_{2}O\right)}=0.08,\;\;\lambda_{H_{2}O}=0.85.$$

The noticeable deviations of Equation (5) from the experimental data take place only in the narrow vicinities of their critical points. As seen from Equation (6) and Figure 5(a), the relative value of the H-bond contribution does not exceed several percents in comparison with that caused mainly by the dispersive interactions.

Thus, the heats of evaporation for normal and heavy water with good accuracy have argon-like behavior. Such a character of the temperature dependencies is also inherent for their specific volumes. These facts give us a reason to assert that the crudest thermodynamic properties of normal and heavy water are formed by the averaged intermolecular potentials. This self-averaging is a result of the rotational motion of water molecules.

**Figure 6.**
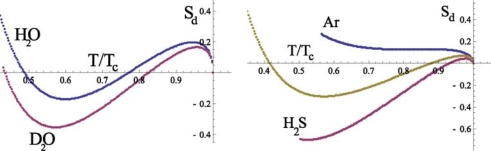
The behavior of the entropy diameter for normal and heavy water, according to the experimental data [28,29].

Now we consider the diameter of the entropy:

$$S_{d}^{\left(w\right)}=\frac{S_{\upsilon }^{\left(w\right)}+S_{l}^{\left(w\right)}}{2S_{c}^{\left(w\right)}}-1,$$

where $S_{c}^{\left(w\right)}$ is the value of the entropy at the critical point, which is the finer thermodynamic characteristics of a system in comparison with *q*^(^*^w^*^)^ (*t*). It characterizes the degree of asymmetry of the vapor and liquid branches of the entropy for water. The behavior of $S_{d}^{\left(w\right)}$ for normal and heavy water (*D*_2_*O*) is presented in Figure 6. For the comparison the entropy diameter for argon as well as for the water homologues *H*_2_*S* and molecular oxygen O_2_ are also presented in Figure 6. We see that the behavior of $S_{d}^{\left(H_{2}O\right)}(t)$is qualitatively different from $S_{d}^{\left(Ar\right)}(t)$. Unlike the latter, the temperature dependence of $S_{d}^{\left(H_{2}O\right)}(t)$ is not monotone and $S_{d}^{\left(H_{2}O\right)}(t)$ vanishes in two points:

$$\begin{array}{c} T_{h}^{\left(H_{2}O\right)}\approx 0.78\cdot 648.7\approx 506K\Rightarrow 233{}^{\circ}C\\ T_{s}^{\left(H_{2}O\right)}=0.484\cdot 648.7=314.3K\Rightarrow 41.2{}^{\circ}C \end{array}$$

The corresponding points for heavy water are:

$$\begin{array}{l} T_{h}^{\left(D_{2}O\right)}\approx 0.78\cdot 648.7\approx 506K\Rightarrow 233{}^{\circ}C\\ T_{s}^{\left(D_{2}O\right)}=0.438\cdot 648.7=282.3K\Rightarrow 9{}^{\circ}C \end{array}$$

They divide the temperature region into three intervals, in which the peculiarities of the entropy are determined by the different physical mechanisms.

From Figure 6 it follows (see details in [30]) that qualitatively such a behavior of the entropy diameter is also characteristic for the systems N_2_, O_2_, F_2_ on their coexistence curves. Therefore, in accordance with our analysis of the heat of evaporation, we should conclude that the appearance of the negative part for the curve $D_{s}^{\left(w\right)}(t)$ in the range *T_s_* *< T < T_h_* is connected with the rotational motion of water molecules. The rapid variation of $D_{s}^{\left(H_{2}O\right)}(t)$ and $D_{s}^{\left(D_{2}O\right)}(t)$ near *Ts* is naturally explained by the reduction of the rotational degrees of freedom in the liquid state of a system when temperature decreases. It is clear that the character of the rotation depends on the number of H-bonds connecting a molecule with its nearest neighbors. Therefore, the change of the rotational motion takes place at the same temperature interval, which is characteristic for the translational motion of molecules.

The further discussion of the rotational motion of molecules and its influence on the thermodynamic properties of water is contained in [31].

## 5. Lower pH and pD Limits for Normal and Heavy Water in the Physiological Temperature Range

It is well known that the strong regulation of the concentration of hydrogen ions in the intercellular and extracellular fluids, and as a result, the balancing pH in body fluids is important for physiological processes such as digestion, hormonal production and others. Optimally, the fluids in our bodies should have a proper pH level. For instance, the normal pH of blood is 7.35 – 7.4. [32]. Any slight variation results in symptoms and disease. For example, if blood pH drops below 6.8, cells stop properly functioning and the patient dies [32].

There is another reason, related to the therapy for cancer treatment, of why it is important to consider the properties of water in the physiological pH range. For example in [33], low pH therapy has been proposed for treating cancer cells. The general idea is to heat the cancer tissues to the a temperature of 42.5 °C and in the same time inject glucose into the blood stream. In the latter case, the cancer cells are starting to live in the acidity environment and going to die at pH = 5.5 or less.

Here we take into account the temperature dependence of pH in pure normal water [34]. As was noted above, the destruction of the H-bond network at *T* > *T_H_* = 315*K* in separated clusters of water molecules such as dimers and trimers occurs. This means that H-bonded water chains could not serve anymore as *H*^+^/*OH*^−^ transfer channels in/out of cells. As a result, the concentration of the hydrogen ions at the temperature *T_H_* = 315*K* takes the critical value for the proper functioning of cells exists. At this concentration, the proper regulation of *H*^+^/*OH*^−^ ions in/out cells is broken and cells start to live in an acidic environment with higher concentration of hydrogen ions and, as a result, are going to die. This means that there is a lower lethal pH limit for the life of warm-blooded organisms.

**Figure 7.**
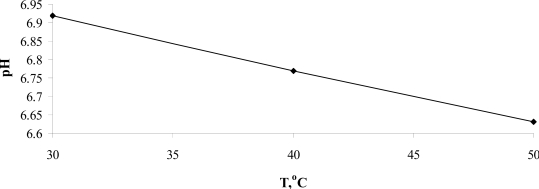
The temperature dependence of pH for the normal water.

In the temperature range 30 °C ≤ T ≤ 50 °C, the temperature dependence of pH, presented in Figure 7, is approximated by the equation:

(7) pH(T)=7.355−9.396·t,

which leads to *pH* (*T_H_*) = (6.747 ∓ 0.044)). This value is close to the unsafe pH one, which equals to 6.8.

One assumes that the value of *pH* = 6.747 could be related to the lower lethal pH limit for functioning cells of warm-blooded organisms. Nevertheless, to prove this fact more research must be conducted.

**Figure 8.**
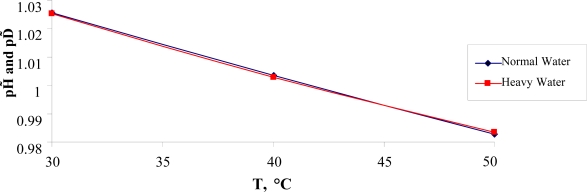
Temperature dependencies of *pH͂* and *pD͂*.

In conclusion of this section, it is important to note the following. In [35] it has been assumed that heavy water in small concentrations could be considered as a possible health cure. The comparative behavior of the normalized values of the degree of the self-ionization, *pH̃* = *pH* (*T*)/*pH* (*T_C_*) and *pD̃* = *pD*(*T*)/*pD*(*T_C_*), for normal and heavy water are presented in Figure 8.

As see from Figure 8, in the temperature range 30°*C* ≤ *T* ≤ 50°*C* the ratio:

$$\frac{p\tilde{H}}{p\tilde{D}}≅1≅inv$$

is invariant of temperature and could be used in further research. Here it is essential to note that heavy water self-ionizes less than normal water, owing to a slightly stronger hydrogen bond.

Using the data taken from [34] it is not difficult to show that the following estimate for heavy water takes place:

(8) $$pD(T_{D})=(7.170\mp 0.045))$$

and it differs from the one for normal water.

One can assume that the value of *pD*(*T_D_*) = 7.170 could be related to the lower lethal pD limit for the life of warm-blooded organisms. We think that this estimate will be useful for developing a new kind of cures (see [35]).

## 6. Temperature Anomalies in the Behavior of the Isothermic Elasticity Modulus and the Entropy Diameter

**Figure 9.**
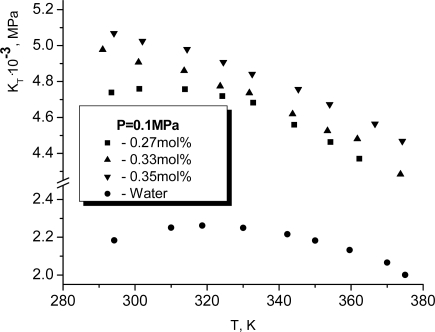
The temperature dependence of the isothermic elasticity modulus for glycerol and water at atmospheric pressure [10].

Isothermic elasticity modulus is determined as:

$$K_{T}\equiv \frac{1}{\beta_{T}^{\left(w\right)}}=-V{\left(\frac{\partial p}{\partial V}\right)}_{T}=\rho {\left(\frac{\partial P}{\partial \rho }\right)}_{T},$$

where *V* and *P* are the volume and the pressure for a system, *ρ* is its mass density. In fact, *K_T_* is the inverse isothermic compressibility. It is well known that *K_T_* for pure water has a maximum near *T_K_* ≈ 318*K*. For *T* > *T_K_* the temperature dependence of the isothermic elasticity modulus becomes argon-like, i.e. it decreases when temperature increases. It is very surprising that the dissolution of glycerol changes such a character of the temperature dependence only if the mole concentration of glycerol exceeds approximately 0.3. At smaller concentrations of glycerol the maximum for *K_T_* continues to be observed. More exactly, it shifts weakly to the left. In particular for *x* = 0.27, as it follows from Figure 9, the respective maximum is observed at *T_K_* ≈ 315*K*.

In the Supplement 2, it is shown that the isothermic compressibilities of water and argon are connected by the relation:

(9) $$\beta_{T}^{\left(w\right)}(t)\approx \beta_{T}^{\left(w\right)}(t)+8\frac{Z}{P_{c}}{\tilde{\upsilon }}^{\left(w\right)}(t)\left(\frac{1}{t}-\lambda \right).$$

where *Z* is the regularized value of the compressibility factor ( $Z=\upsilon_{R}^{\left(w\right)}P_{c}/k_{B}\;T$) and *P_c_* is the critical pressure. Thus, the isothermal compressibility of water is the combination of two terms having different character of the temperature dependencies: the first term in Equation (9) increases with the temperature *t* = *T* / *T_c_*, where *T_c_* is the critical temperature, and the second one – diminishes when temperature grows. Therefore, the prerequisite for the non-monotone temperature dependence of the isothermic compressibility of water arises. The numerical analysis of Equation (9) shows that the minimum for $\beta_{T}^{\left(w\right)}$ is observed near its experimental value *T_β_* ≈ 318*K*.

Although *T_β_* is rather different from *T_n_*, *T_ν_* and *T_s_*, they all are connected with the temperature dependence of the same characteristics of the H-bond network - the averaged number of H-bonds per molecule. Therefore, *T_β_* can be considered as an independent estimate for *T_H_*.

Note that the similar argumentation is also applicable for the water glycerol solutions. From here, it follows that the maintenance of the non-monotonous temperature dependence of the compressibility in the water-glycerol solutions allows us to hope that the similar manifestation of H-bonds will take place in the intracellular and extracellular fluids.

**Figure 10.**
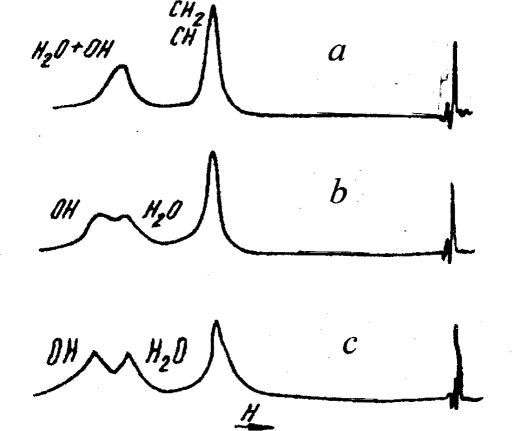
Spectra of ^1^*H* – *NMR* for the water-glycerol solution with the mole concentration *x*=0.51 of glycerol at different temperatures: *a* – 308*K*, *b*=283*K*, *c*–273*K*, according to [11].

A very important addition to these results for the water-glycerol solutions is given by the NMR investigations presented in [11]. It was observed that the lines corresponding to water molecules and *OH* -bonds at *T* > 308*K* interflow to the one peak. At the same time, at *T* < 308*K* they are separated – a peak has the doublet structure (Figure 10).

Since the peculiarities of the NMR resonance depend on the character of the thermal motion of molecules, i.e. on the ordering degree of the H-bond network, we should conclude that the NMR indicate this change at *T_NMR_* = 308*K*.

## 7. Self-Diffusion of the Water Molecules in Water-Electrolyte Solutions

There are all reasons to suppose that the thermal motion of ions in cells has similar traits with those in water-electrolyte solutions, whose properties are essentially simpler. Therefore, in this Section we will consider the peculiarities of the diffusion motion of water molecules in the water-electrolyte solutions for which there are numerous experimental data, obtained by quasi-elastic incoherent neutron scattering.

We expect that the thermal motion of water molecules in the water-electrolyte solutions also has crystal-like character, since the electric field of ions, similarly to H-bonds, holds water molecules in their vicinity. It is necessary to take into account that the temporary equilibrium positions of water molecules can be in water bulk or in the hydrate shells of ions. In correspondence with this, we differ the residence times $\tau_{0}^{\left(w\right)}$ and $\tau_{0}^{\left(h\right)}$, which are dependent on the ion concentrations. It seems evident that for the dilute water-electrolyte solutions the diffusion peak is mainly formed by molecules from the bulk phase. Therefore, the half-width of the diffusion peak is equal to:

(10) $$\gamma_{D}({\vec{k}}^{2})\approx D_{s}{\vec{k}}^{2}-\tau_{0}^{\left(w\right)}D_{s}^{{\left(1\right)}^{2}}{\vec{k}}^{4}+\tau_{0}^{{\left(w\right)}^{2}}D_{s}^{{\left(1\right)}^{3}}{\vec{k}}^{6}+\ldots ,$$

where all designations are similar to those in Equation (2). In the opposite case, when practically all water molecules are in the hydrate shells, the formula (10) transforms to:

(11) $$\gamma_{D}({\vec{k}}^{2})\approx D_{s}{\vec{k}}^{2}-\tau_{0}^{\left(h\right)}D_{s}^{{\left(1\right)}^{2}}{\vec{k}}^{4}+\tau_{0}^{{\left(h\right)}^{2}}D_{s}^{{\left(1\right)}^{3}}{\vec{k}}^{6}+\ldots .$$

The numerical values of all parameters in Equations (10) and (11) can be found by fitting the experimental data on the half-widths $\gamma_{D}({\vec{k}}^{2})$ of the diffusion peaks for the quasi-elastic incoherent neutron scattering, considering them as a function of ${\vec{k}}^{2}$. Unfortunately, experimental data for dilute water-electrolyte solutions are absent in the literature. Thus, the concentrations of *LiCl*, *NaCl* and *KCl* in [36–38] take values *z_w_* = 27.8, 13.9, 6.05 that is essentially greater than for the intracellular and extracellular fluids (here *z_w_* denotes the number of water molecules per ion). For the water solution of the table salt *NaCl*, the numerical values of all parameters entering Equation (9) are presented in Table 1:

Here $r_{c}^{+}$ is the crystallographic radius of the *Na*^+^ -ion. For pure water, the corresponding parameters take the following values [15,16]:

$$D_{s}=2.2\cdot 10^{-5}cm^{2}\;/\;s,\;\;D_{c}=0.17\cdot 10^{-5}cm^{2}/s,\;\;\tau_{0}=0.8\cdot 10^{-12}s.$$

As seen in Table 1, the addition of electrolyte (*z_w_* diminishes) leads to the increasing of the self-diffusion coefficient for water molecules. The residence time of them remains to be practically invariable at the low concentrations. At the same time, the contribution caused by the collective drift of molecules increases twice in comparison with pure water. This circumstance is especially important for the intracellular fluid where the collective effects play especially an important role.

The manifestation of the collective effects is especially relief for the concentrated water-electrolyte solutions (*z_w_* = 6.05) [39]. At that for the water solution of *LiCl* the temperature dependence of *D_c_* remains to be monotone: only the character of this dependence is changed near *T* ~ *T_H_*. For the water solutions of the table salt *NaCl* and *KCl* the corresponding variations of the temperature dependencies for *D_c_* are essentially more considerable.

Unfortunately, the experimental data for these dilute solutions are absent. It seems to be natural to assume that at the concentration *z_w_* = 6.05 practically all water molecules belong to the first coordination shell of ions. Therefore, the collective drift of water molecules in such solutions should be close to the self-diffusion coefficients of ions. This assumption is qualitatively consistent with the values of the ion radii given in Table 2.

From Table 2 it follows that also the residence time of water molecules radically increases with concentration. It leads to the considerable growth of the parameter $l_{0}=\sqrt{D_{s}\tau_{0}}$. From Tables 1 and 2, we find: $l_{0}^{\left(low\right)}$ ≈ 0.4 · 10^−8^ *cm* and $l_{0}^{\left(high\right)}$ (*NaCl*) ≈ 1.8 · 10^−8^ *cm*. The latter value is close to the diameter of the ion *Na*^+^. In the first case, the value of $l_{0}^{\left(low\right)}$ is consistent with that displacement of a water molecule, which corresponds to the bending of two H-bonds [2,4].

## 8. Average Number of H-Bonds Per Molecule in Bulk Water

In this Section we briefly consider the general method for the determination of *n_H_* (*t*) as a function of temperature. A key role in this approach belongs to the comparative analysis of specific volume per molecule for water and argon in the manner of the principle of corresponding states.

**Figure 14.**
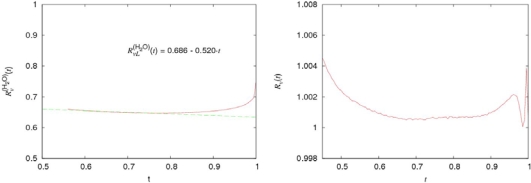
Temperature dependencies of the ratios $R_{\upsilon }^{\left(H_{2}O\right)}(t)$ = υ*^(H^*^_2_^*^O^*^)^*(t)*/*υ^(Ar)^(t)* and *R_υ_*(*t*) *= υ*^(^*^D^*^_2_^*^O^*^)^*(t)*/*υ*^(^*^H^*^_2_^*^O^*^)^*(t)* on the coexistence curves of normal and heavy water, and argon, according to [24,25].

The comparison of the temperature dependencies of the specific volume per molecule *υ*^(^*^i^*^)^ for normal water and argon (*i* = *H*_2_*O*, *Ar*) and *R_υ_* (*t*) *=* *υ^(D^*^_2_^*^O)^(t)*/*υ^(H^*^_2_^*^O)^* *(t)* is presented in Figure 14. The dimensionless temperature $t=T/T_{C}^{\left(i\right)}$, where $T_{C}^{\left(i\right)}$ is the critical temperature of liquids, is used.

As seen, the specific volumes of normal and heavy water demonstrate very surprising peculiarities. Practically in the whole region of liquid states the behavior of *υ*^(^*^i^*^)^, *i* = *H*_2_*O*, *D*_2_*O* is argon-like. Only in the narrow vicinity of the critical point (0.95 < *t* < 1), the deviation from the argon-like dependence is essential. In the rest of the region (*t_m_* < *t* < 0.90), where *t_m_* is the melting point (*t_m_* = 0.42 for normal water), the deviation from the argon-like dependence does not exceed (3 ÷ 4)%. In accordance with Figure 14, the temperature dependence of $R_{\upsilon }^{\left(H_{2}O\right)}(t)$ can be approximated as:

(12) $$R_{\upsilon }^{\left(H_{2}O\right)}(t)=0.63+r_{H}(t).$$

In accordance with what was said above, the contribution *r_H_* (*t*) is caused by H-bonds and its value is smaller than (0.03 ÷ 0.04) in the temperature interval 0.5 < *t* < 0.9.

For description of the temperature dependence of *r_H_* (*t*) seems to be natural to apply Hilbert’s principle, which was formulated for the first time in the algebraic invariant theory [40] and which has the numerous applications in the statistical hydrodynamics [41]. According to this principle, an arbitrary complicated function can be expanded in the series with respect to independent primitive functions, which have the same properties of symmetry. In particular, for water a role of the primitive functions {*S_i_*} should play the independent structural characteristics of the H-bond network, so-called structural functions [26,27]. Thus:

(13) $${\tilde{\upsilon }}_{H}^{\left(H_{2}O\right)}(t)=\sum_{k}\upsilon_{k}^{\left(H\right)}\cdot S_{k}(t,p),$$

The most important structural functions are the average number *n_H_* of H-bonds per molecule and the parameter of the tetrahedricity χ (see [42,43]). The structural functions of a higher order are assumed to be responsible for the finer details of the H-bond network, and here they will be ignored. In regard to *r_H_* (*t*), with good accuracy, it can be approximated only by the contribution of *n_H_* [26,27]:

(14) $$r_{H}(t)=r_{H}^{\left(0\right)}n_{H}(t)+\ldots ,$$

where $r_{H}^{\left(0\right)}=0.015$ and:

(15) $$n_{H}(t)=4(1-0.85\cdot t).$$

From Equation (12) it follows that:

(16) $$\upsilon^{\left(H_{2}O\right)}(t)=0.63\cdot \upsilon^{\left(Ar\right)}(t)+r_{H}^{\left(0\right)}\cdot \upsilon^{\left(Ar\right)}(t)\cdot n_{H}(t).$$

Since the first term in Equation (16) increases with temperature and the second one have the opposite behavior, the formula (16) naturally explains the appearance of the minimum of *υ*^(^*^w^*^)^ (*t*) near *T_υ_* ≈ 277*K*.

The estimates for *n_H_* (*t*) very close to Equation (15) follows also from the analysis of the heat of evaporation in Section 4 and the kinematic shear viscosity in [21,22,27], where they are also obtained with the help of 1) the principle of corresponding states; and 2) Hilbert’s principle. Practically the same results were represented in [44] from the study of the heat capacity. We would like to draw attention to the estimates for *n_H_* (*t*) obtained in the last years from the careful analysis of the

temperature dependencies of the dielectric permittivity at the room temperatures in [45] and the X-ray scattering in [46]. The numerical values of *n_H_* (*t*) obtained with the help of the computer simulations are rather greater [47,48] than ones obtained by us. This difference is connected with the specificity in the definition of H-bonds [48].

Note that the consideration of the association process, presented in [49], leads to the conclusion that liquid water is the ensemble of dimers in the fluctuation region. The practically full dimerization of water molecules allows us to explain naturally the sharp enough increment of the ratio *R_υ_* near the critical point (see Figure 14).

## 9. Conclusions

In this paper, the main attention has been focussed on those peculiarities of the thermal motion in water as well as in the water-electrolyte and water-glycerol solutions, which are characteristic for the temperature interval of the life for warm-blooded organisms, i.e. for 300*K* < *T* < 315*K*.

Analyzing: 1) the angular dependence of the half-width for the diffusion peak in the quasi-elastic incoherent neutron scattering in water; 2) the behavior of the kinematic shear viscosity of water in the whole range of its liquid states; and 3) the temperature dependence of the diameter of entropy, it is shown that the character of the thermal motion in water undergoes an essential change at the temperature *T_H_* ≈ (315 ± 3)*K*. The peculiarities of the thermal motion discovered in such a way were completed by the analysis of the temperature dependencies for the specific volume and the heat of evaporation per molecule. All the facts stated in this paper allows us to conclude that near the characteristic temperature *T_H_*:

1. the global H-bond network disintegrates on the ensemble of weakly interacting clusters, in the first place: dimers, trimers, tetramers and so on;
2. the crystal-like character of the thermal motion at *T* ~ *T_H_* transforms to the argon-like one;
3. the relation between the characteristic times *τ_r_* for the rotational motion and $\tau_{s}∼\sqrt{a\;/\;\upsilon_{T}}$ for the soft collisions of molecules (see [50]) changes in the following way: *τ_r_*/*τ_s_* > 1 for *T* < *T_H_* and *τ_r_*/*τ_s_* < 1 for *T* > *T_H_*.

All these peculiarities of the thermal motion are tightly connected with each other since they are determined by the formation of H-bonds between molecules. In the present work it is shown that the average number of H-bonds per a molecule near *T* ~ *T_H_* takes the value *n_H_* (*T_H_*) ≈ 2.34 that is close to *n_H_* = 2, which corresponds to the ensemble of the linear molecular chains. We expect that the spatial connectivity between them is violated near *T* ~ *T_H_*. In other words, we suppose that considerable fluctuations of the spatial connectivity for linear chains take place near *T* ~ *T_H_*. The temperature interval inside which this transformation takes place does not exceed ten degrees. Therefore, this assumption and all facts enumerated above form basis for the assertion that *T_H_* is the temperature of the dynamic phase transition. We emphasize that all thermodynamic and kinetic quantities change monotone at *T* ~ *T_H_*. Only the character of the thermal motion changes considerably near this temperature.

Based upon these facts, the upper death temperature limit for warm-blooded organisms can be defined as the temperature at which intracellular water is undergoing to the dynamic phase transition, i.e. the character of the thermal motion transforms from crystal-like to argon-like and the ordering degree of the H-bond network essentially changes. In accordance with this definition, the dynamic phase transition is considered as a necessary prerequisite for the denaturation of proteins inside cells. This circumstance is undervalued in the standard approaches [52,53].

Thus, the physiological temperature range for the warm-blooded organisms corresponds to the following conditions:

1. the existence of the fragile global H-bond network, for which *n_H_* ~ (2.2 ÷ 2.3);
2. the concentrations of bio-inclusions in cells should not exceed 15 mol % that is approximately half of the maximal value, leading to the suppression of the water properties;
3. pH satisfies the inequality: *pH* > 6.8.

These conditions guarantee the existence of comparatively small shear viscosity and the large enough values of the dielectric permittivity, which are necessary for the normal energy-, mass-, and ions exchange in cells.

Since the difference between maximal and minimal values of *T_n_*, *T_ν_*, *T_s_* and *T_β_* is 8*K*, the conclusion about the smeared dynamic phase transition at *T_H_* ≈ 315*K* seems to be quite justified. In the case of heavy water, the situation is not so definitive. The difference $T_{v}^{\left(D_{2}O\right)}-T_{s}^{\left(D_{2}O\right)}\approx 15K$ is twice more than for normal water. From here it follows that the different properties for heavy water in the vicinity of the dynamic phase transition change discordantly. It seems that this circumstance essentially impedes to the normal functioning of proteins in cells filled by heavy water. Unfortunately, a detailed study of the thermal motion in heavy water with the help of the quasi-elastic incoherent neutron scattering is lacking. In connection with this problem, one can put a question about the synthesis of proteins in which H-bonds are replaced by D-bonds.

The maintenance of the non-monotone temperature dependence for the isothermal compressibility or the isothermal elasticity modulus in the water-glycerol solutions at high enough concentrations, up to *x* = 0.27 mole fractions, is especially surprising. In the weight fractions this concentration limit for glycerol is about 0.7 that exceeds by more than twice the concentration of bio-inclusions in a cell. This fact allows us to suppose that the behavior of bio-inclusions in cells is substantially determined by the properties of bulk water. In connection with this, it is necessary to note that the intracellular fluid in a cell is usually separated into bulk and biological (surfacial) water [54]. This circumstance, as it is clear, takes also place in the concentrated enough water-glycerol solutions; therefore, we should conclude that it is not crucial. From this point of view, the disappearance of the doublet splitting of the NMR-peak at *T* > *T_H_* is not occasional. It is naturally explained by the change of the character of the thermal motion at *T* ~ *T_H_*. Of course, the fine manifestation of the effects of the confinement geometry should be taken into account.

The additional important information about the role of ions in cells is given by the quasi-elastic incoherent neutron scattering in the water electrolyte solutions. Here, as we have seen, the collective drift of water molecules and the residence time undergo the most essential changes. It is very surprising that the temperature ranges for the non-monotone behavior of the self-diffusion coefficients for water molecules in the water - *NaCl* and water - *KCl* solutions are close to the life temperature range for warm-blooded organisms. Although the nature of this coincidence is not clear now, it is scarcely occasional. The self-diffusion coefficient of the water molecules is also changed but this effect is not so clearly marked. The concentration of ions in the intracellular and extracellular fluids [55] is smaller by several digits in comparison with that in the laboratory experiments. In this case, the changes described above will manifest themselves locally that is probably very important.

The different kinds of ions in the intracellular and extracellular fluids play very important role in the pH regulations for the proper functioning of cells of warm-blooded organisms. Using our estimates one can suppose that the lower physiological pH limit for the life of warm-blooded organism, which corresponds to *pH*(*T_H_*), is equal to 6.8. To restore a pH balance in the body fluids to the normal one, the addition of ions is necessary. In the live organism, this process, which is called acid-base homeostasis, has a self-regulatory character. It follows to expect that near *T_H_* the self-regulations of pH become to be hampered and the external regulation should be applied.

The H-bonds play the essential role in the formation of properties of the intracellular fluid and proteins interacting with it. Therefore, the integrity of the H-bond network in the body fluids should considerably effect on the protein denaturation.

Our consideration shows that near *T_H_* the character of the thermal motion essentially changes in the pure bulk water as well as in the water-glycerol and water-electrolyte solutions. In connection with this, we expect that similar influence of the H-bond network will also take place in both the intracellular and extracellular fluids. From this point of view, the characteristic temperature *T_H_* can be interpreted as the upper temperature limit for the life of warm-blooded organisms. It is not excluded that the week dependence of *T_H_* on concentrations of salts or glycerol in cells is manifested in the higher values of death temperatures for birds [56].

It is essential to note that the different organs of warm-blooded organisms have unequal death temperatures. For instance, the working temperature of the human liver is *T_w_* ~ 315*K*. The main function of the liver is the purification of blood. The effectiveness degree of this process depends immediately on the integrity of the H-bond network in the blood plasma. The separation of impurities is facilitated when the influence of H-bonds becomes weaker. Namely, near *T_w_* ≈ *T_H_* the integrity of the H-bond network in the blood plasma is destroyed and we can expect that the mobility of impurities increase. From physical point of view, it depends not only upon the protein composition of a tissue, but also upon the character of the H-bond network in the surrounding intracellular and extracellular fluids.

The destruction of the H-bond network in the intracellular fluid of the blood cells as well as in the extracellular fluid of plasma at *T* → *T_H_* is probably accompanied by the reduction of the solubility for oxygen and nutrients and also the ability to carry waste materials away from the brain tissues for disposal. As a result, the lack of oxygen in the brain tissue leads to its death (it is supposed that the brain is the most sensitive to the oxygen nourishment). Unfortunately, we cannot confirm this conjecture since the experimental data on the solubility of oxygen in blood as a function of temperature are not known to us.

A general concept developed in this paper has direct applications to genetics and bacterial biophysics. In these cases, the structural changes in water and aqueous solutions, especially in the range of 40–43°C, influence the behavior or activity of biological systems. Some examples are: a) the conditional lethal mutations within a single cell [57]; b) the multiple temperature optima for the growth of the organism of bacteria [58]. For instance, a colony of *E. coli* fails to grow at 42 ºC.

In conclusion, it is also essential to note that special attention should be directed to the attentive investigation of the thermal motion in normal and heavy water near the lower physiological temperature limit for the life of warm-blooded organisms. This analysis will also be incomplete without a research related to the determination of the upper physiological pH limit. These and other topics will be points of discussion in subsequent publications.

## Supplements

Supplement 1. The Two Differential Cross-Sections for the Quasi-Elastic Incoherent Neutron Scattering Let us construct the differential equation for the intermediate scattering function [59, 60]:
(17)$F_{s}(\vec{k},t)=<e^{i\vec{k}\cdot \Delta \vec{r}(t)}>\;,$which could be corresponding to the picture of the thermal motion presented in the Section 2. In accordance with this picture, one can write:
(18)$$F_{s}(\vec{k},t_{N})=<\;\exp(i\vec{k}{\left(\Delta {\vec{r}}_{1}+\Delta {\vec{r}}_{2}+\ldots +\Delta {\vec{r}}_{N}\right)}^{2})>\;\;,$$where *t_N_* = *Nτ*, $\tau =\tau_{0}+\tau_{1}$, $\Delta {\vec{r}}_{i}$ is the displacement of a molecule in time (*i* − 1)*τ* < *t* < *iτ*. The every contribution in Equation (18) is the sum of two terms:
$\Delta {\vec{r}}_{i}(t)=\Delta {\vec{r}}_{i}^{\left(o\right)}(t)+\Delta {\vec{r}}_{i}^{\left(d\right)}(t),$where the first of them corresponds to the oscillation motion and the latter one to the irreversible thermal drift from the one temporary equilibrium position to another. The maximum value of the oscillation amplitude is essentially smaller than the interparticle spacing *a*:
(19)$$\max(\Delta {\vec{r}}_{i}^{\left(o\right)}\;(t))\;<<a,$$and the characteristic oscillation frequencies $\omega_{k}^{\left(0\right)}$ are essentially greater than the typical inverse time 1 / *τ* of the drift motion:
$$\min(\omega_{k}^{\left(0\right)})>>1/\tau .$$
Due to this, the averaging on the oscillation and the drift motions of a molecule can be produced independently:
$$F_{s}(\vec{k},t_{N})=<\exp\left(\sum_{i=1}^{N}i\vec{k}\cdot \Delta {\vec{r}}_{i}^{\left(o\right)}(t)\right){>}^{\left(o\right)}<\exp\left(\sum_{i=1}^{N}i\vec{k}\cdot \Delta {\vec{r}}_{i}^{\left(d\right)}(t)\right){>}^{\left(d\right)}$$Since the oscillation amplitudes are obeyed to the inequality (19), for the long wave limit ( $|\vec{k}|a<<1$), we obtain:
(20)$$<\exp\left(\sum_{i=1}^{N}i\vec{k}\cdot \Delta {\vec{r}}_{i}^{\left(o\right)}(t)\right){>}^{\left(o\right)}\Rightarrow \exp(-2W),$$where exp(−2*W*) with $2W=\frac{1}{6}{\vec{k}}^{2}<{\left(\Delta {\vec{r}}_{i}^{\left(o\right)}(t)\right)}^{2}{>}^{\left(o\right)}\equiv \frac{1}{6}{\vec{k}}^{2}A^{2}$, is the Debye-Waller factor [59]. Thus,
(21)$$F_{s}(\vec{k},t_{N})\Rightarrow \exp(-2W)F_{d}(\vec{k},t_{N}),$$where:
(22)$$F_{d}(\vec{k},t_{N})=<\exp\left(\sum_{i=1}^{N}i\vec{k}\cdot \Delta {\vec{r}}_{i}^{\left(d\right)}(t)\right){>}^{\left(d\right)}.$$
Supposing that the two successive displacements $\Delta {\vec{r}}_{i-1}^{\left(d\right)}$ and $\Delta {\vec{r}}_{i}^{\left(d\right)}$ are uncorrelated, the function $F_{d}(\vec{k},t_{N})$ can be represented as
$$F_{d}(\vec{k},t_{N})=F_{d}(\vec{k},t_{N-1})f_{N}({\vec{k}}^{2}),\;.f_{N}({\vec{k}}^{2})=<\exp(i\vec{k}\Delta {\vec{r}}_{N}^{\left(d\right)})\;>,$$where the angular brackets denote the averaging over the directions of $\Delta {\vec{r}}_{N}$ on the unite sphere. Starting from this, we can construct the following approximate equation for the intermediate scattering function $F_{s}(\vec{k},t)$:
(23)$$\frac{\partial F_{d}(\vec{k},t)}{\partial t}\approx \frac{1}{\tau }\left(F_{d}(\vec{k},t_{N})-F_{d}(\vec{k},t_{N-1})\right)\Rightarrow \frac{1}{\tau }F_{d}(\vec{k},t_{N-1})(1-f_{N}({\vec{k}}^{2})),\;N>>1.$$
The simplified form of such a construction was considered in [60]. In general, the displacement $\Delta {\vec{r}}_{N}$ of a molecule during the time *τ* can be represented as the sum:
$$\Delta {\vec{r}}_{N}^{\left(d\right)}=\Delta {\vec{r}}_{N}^{\left(c\right)}+\;\Delta {\vec{r}}_{N}^{\left(1\right)},$$where the first term $\Delta {\vec{r}}_{N}^{\left(c\right)}$ describes the collective drift of a molecule in the field of the thermal hydrodynamic fluctuations and the second one $\Delta {\vec{r}}_{N}^{\left(1\right)}$ - the displacement of a molecule relatively its nearest neighbors. Both types of these displacements are independent since the first of them is caused by the low-frequency modes in a system and the latter one by the high frequency ones. Due to this, we can write:
$$f_{N}({\vec{k}}^{2}))=f_{N}^{\left(c\right)}({\vec{k}}^{2})\cdot f_{N}^{\left(1\right)}({\vec{k}}^{2}),$$where:
$$f_{N}^{\left(c\right)}({\vec{k}}^{2})=<\exp(i\vec{k}\Delta {\vec{r}}_{N}^{\left(c\right)})>,\;f_{N}^{\left(1\right)}({\vec{k}}^{2})=<\exp(i\vec{k}\Delta {\vec{r}}_{N}^{\left(1\right)})>.$$
The collective drift is continuous for all times noticeably larger them the characteristic molecular time *τ_s_*. Thus, the distribution function for $\Delta {\vec{r}}_{N}^{\left(c\right)}$ should take the Gaussian form:
$W_{G}\left(\Delta {\vec{r}}_{N}^{\left(c\right)}(\tau )\right)={\left[4\pi D_{c}\tau \right]}^{-3/2}\;\exp\left(-\frac{{\left(\Delta {\vec{r}}_{N}^{\left(c\right)}(\tau )\right)}^{2}}{4D_{c}\tau }\right),$where *D_c_* is the collective part of the self-diffusion coefficient. For such a distribution
$$f_{N}^{\left(c\right)}({\vec{k}}^{2})=\exp(-6D_{c}\tau ).$$
Note that the collective part of the self-diffusion coefficient was first introduced in [61]. The importance of this notation for the physics of liquid was motivated in [62,63]. The methods of calculations of *D_c_* have been developed in [62–64]. The careful determination of *D_c_* at different temperatures for water is given in [5].
It is not difficult to verify that after the averaging on the angular variables, the function:
$$f_{N}^{\left(1\right)}({\vec{k}}^{2})=〈\frac{\sin k\left|\Delta {\vec{r}}_{N}^{\left(1\right)}\right|}{k\left|\Delta {\vec{r}}_{N}^{\left(1\right)}\right|}〉.$$
Here the angular brackets denote the averaging with the distribution function $p(\left|\Delta {\vec{r}}_{N}^{\left(1\right)}\right|)$. Unlike $\Delta {\vec{r}}_{N}^{\left(c\right)}$ the distribution function for $\Delta {\vec{r}}_{N}^{\left(1\right)}$ has another nature. During the characteristic time *τ*_1_ ≪ *τ*_0_, a molecule displaces about the nearest neighbors on the distance ~ *l*_0_ ≤ *a*, where *a* is the interparticle spacing. On the time scales of order *t* ~ *τ*, this type of the motion can be considered as a jump-like with:
$$p(\left|\Delta {\vec{r}}_{N}^{\left(1\right)}\right|)=\frac{\left|\Delta {\vec{r}}_{N}^{\left(1\right)}\right|}{l_{0}^{2}}\exp\left(-\frac{\Delta {\vec{r}}_{N}^{\left(1\right)}}{l_{0}}\right).$$
After the averaging with this distribution function, we find:
$$f_{N}^{\left(1\right)}({\vec{k}}^{2})=\frac{1}{1+{\vec{k}}^{2}l_{0}^{2}}.$$
Taking into account this result and then substituting $F_{d}(\vec{k},t)$ in Equation (23) instead of $F_{d}(\vec{k},t_{N-1})$, we obtain the final differential equation for the intermediate function $F_{d}(\vec{k},t)$:
(24)$$\frac{\partial F_{d}(\vec{k},t)}{\partial t}=-\frac{1}{\tau }\left(1-\frac{\exp(-\tau D_{c}{\vec{k}}^{2})}{1+{\vec{k}}^{2}l_{0}^{2}}\right)F_{d}(\vec{k},t).$$
The function $F_{d}(\vec{k},t)$ as a solution of Equation (24) is equal to:
(25)$$F_{d}(\vec{k},t)=\exp\left(-\Lambda ({\vec{k}}^{2})t\right),$$where:
(26)$\Lambda ({\vec{k}}^{2})=\frac{1}{\tau }\left(1-\frac{\exp(-\tau D_{c}{\vec{k}}^{2})}{1+\tau D_{s}{\vec{k}}^{2}}\right)$determines the half-width of the peak for the incoherent neutron scattering spectrum [59,60]. Here, the value:
$D_{s}^{\left(1\right)}=\frac{l_{0}^{2}}{\tau }$has the meaning the one-particle contribution to the self-diffusion coefficient, since:
$D_{s}^{\left(1\right)}=\frac{<{\left(\Delta {\vec{r}}_{i}\right)}^{2}>}{6\tau }\Rightarrow \frac{l_{0}^{2}}{\tau }.$The parameter *l*_0_ is often interpreted as the averaged jump of a molecule that is not correct, since the drift on *l*_0_ is carried out during the finite time *τ*_1_ ~ *τ_s_*.
Note that the structure of Equation (26) is close to that:
$\Lambda_{SS}({\vec{k}}^{2})=\frac{1}{\tau }\left(1-\frac{\exp(-2W)}{1+\tau D_{s}{\vec{k}}^{2}}\right),$obtained in [14] on the basis of assumptions, which cannot be justified from the physical point of view. Besides, the influence of the oscillation motion, i.e. the Debye-Waller factor, on the time evolution on the diffusion mode is especially problematic. Our result Equation (26) is free from this shortcoming.
The applicability region for the diffusion approximation is determined by the inequalities: $\tau D_{c}{\vec{k}}^{2}\;\ll 1$ and $l_{o}^{2}{\vec{k}}^{2}\ll 1$. Therefore, the solution of Equation (25) takes the form:
(27)$$F_{d}(\vec{k},t)=\exp\left(-D_{s}({\vec{k}}^{2}){\vec{k}}^{2}t\right),$$where :
(28)$$D_{s}({\vec{k}}^{2})=D_{s}\left(1-\tau D_{s}^{\left(1\right)}{\vec{k}}^{2}+{\left(\tau D_{s}^{\left(1\right)}{\vec{k}}^{2}\right)}^{2}+\ldots \right),$$
The combination $D_{s}=D_{s}^{\left(1\right)}+D_{c}$ has the meaning of the full self-diffusion coefficient. Its value is connected with the half-width for the quasi-elastic incoherent neutron scattering by the trivial relation: $D_{s}=d\gamma_{D}({\vec{k}}^{2})/d{\vec{k}}^{2}{|}_{{\vec{k}}^{2}=0}$.
In accordance with Equations (21) and (27), the two differential cross-sections for the incoherent neutron scattering is equal to:
(29)$${\left(\frac{d^{2}\sigma }{d\Omega d\varepsilon }\right)}_{inc}=\frac{b_{inc}^{2}k}{\pi \hbar k_{0}}\exp(-2W)\frac{\gamma_{D}}{\omega^{2}+\gamma_{D}^{2}},$$where the half-width $\gamma_{D}({\vec{k}}^{2})$ of the Lorentzian is equal to:
(30)$$\gamma_{D}({\vec{k}}^{2})\approx D_{s}\left(1-\tau D_{s}^{\left(1\right)}{\vec{k}}^{2}+{\left(\tau D_{s}^{\left(1\right)}{\vec{k}}^{2}\right)}^{2}+\ldots \right){\vec{k}}^{2},$$
If we take into account that 1) the collective contribution to the self-diffusion coefficient is considerably smaller in comparison with *D_s_* practically in the whole temperature interval of liquid states and 2) in the applicability region of the crystal-like representation the inequality *τ*_1_ ≪ *τ*_0_ takes place, we can simplify Equation (30) in the following way:
(31)$$\gamma_{D}({\vec{k}}^{2})\approx D_{s}{\vec{k}}^{2}-\tau_{0}D_{s}^{{\left(1\right)}^{2}}{\vec{k}}^{4}+\tau_{0}^{2}D_{s}^{{\left(1\right)}^{3}}{\vec{k}}^{6}+\ldots .$$
Using Equation (31) for fitting the experimental data on the quasi-elastic incoherent neutron scattering, we can determine all important parameters: *D_s_*, the residence time *τ*_0_ and the one-particle contribution $D_{s}^{\left(1\right)}$ to the full self-diffusion coefficient or its collective part: $D_{c}=D_{s}-D_{s}^{\left(1\right)}$.
Let us note that the formula (29) leads to the correct influence of the oscillation motion of molecules on the integral intensity of the neutron scattering:
$${\left(\frac{d\sigma }{d\Omega }\right)}_{inc}=\frac{b_{inc}^{2}k}{\hbar k_{0}}\exp(-2W).$$

Supplement 2. Qualitative analysis of the water isothermic compressibility By the definition, the isothermic compressibility for water is described by the standard expression [65]:
(32)$\beta_{T}^{\left(w\right)}=\frac{1}{nk_{B}T}\left[1+4\pi n\int_{0}^{\infty }\left(g^{\left(w\right)}(r)-1\right)r^{2}dr\right].$The contribution of the second term can be represented in the form:
(33)$4\pi n\int_{0}^{\infty }\left(g^{\left(w\right)}(r)-1\right)r^{2}dr=4\pi n\int_{0}^{\infty }\left(g^{\left(Ar\right)}(r)-1\right)r^{2}dr+4\pi n\int_{0}^{\infty }\left(g^{\left(w\right)}(r)-g^{\left(Ar\right)}(r)\right)r^{2}dr$The binary correlation functions *g* ^(^*^w^*^)^ (*r*) and *g* ^(^ *^Ar^*^)^ (*r*) for water and argon essentially differ from each other only in the first coordination spheres. Therefore, with good accuracy we can write:
$4\pi n\int_{0}^{\infty }\left(g^{\left(w\right)}(r)-g^{\left(Ar\right)}(r)\right)r^{2}dr\approx \Delta N_{1},$where Δ*N*_1_ is the difference of the numbers of molecules in the first coordination spheres. In this approximation, the expression Equation (32) allows us to represent the isothermic compressibility for water as:
(34)$$\beta_{T}^{\left(w\right)}(t)=\beta_{T}^{\left(w\right)}(t)-\frac{1}{k_{B}T}\left[\left(\Delta N_{1}-1)\upsilon^{\left(w\right)}(t)+\upsilon^{\left(Ar\right)}(t\right)\right].$$
Near the melting point of water, the value Δ*N*_1_ is negative and noticeably larger than unit; therefore, Equation (34) is simplified:
(35)$$\beta_{T}^{\left(w\right)}(t)\approx \beta_{T}^{\left(w\right)}(t)+\frac{\upsilon^{\left(w\right)}(t)\left|\Delta N_{1}\right|}{k_{B}T}.$$
The first term in Equation (35) increases with temperature, the second one decrease. The temperature dependence of the latter is mainly determined by the multiplier | Δ*N*_1_ | and its value steadily diminishes when the temperature increases.
The second term in Equation (35) can be simplified if we suppose that the number of the nearest neighbors in water is approximated by the linear expression:
$$N_{1}^{\left(w\right)}=4+8\left(1-\frac{1}{4}n_{H}(t)\right),$$in which it supposed that $N_{1}^{\left(w\right)}=12$ in the absence of H-bonds. In argon the nearest neighbors is close to this value. Hence, we have:
$$\left|\Delta N_{1}\right|\approx 2n_{H}(t).$$
Approximating the average number of H-bonds per molecule on the coexistence line with the help of *n_H_* (*t*) ≈ 4(1 − *λt* +...) (see Equations (4) and (15)), we can represent the isothermic compressibility of water in the form:
(36)$$\beta_{T}^{\left(w\right)}(t)\approx \beta_{T}^{\left(w\right)}(t)+8\frac{\upsilon_{R}^{\left(w\right)}}{k_{B}T_{c}}{\tilde{\upsilon }}^{\left(w\right)}(t)\left(\frac{1}{t}-\lambda \right),$$or:
(37)$\beta_{T}^{\left(w\right)}(t)\approx \beta_{T}^{\left(w\right)}(t)+8\frac{Z}{P_{c}}{\tilde{\upsilon }}^{\left(w\right)}(t)\left(\frac{1}{t}-\lambda \right).$is the regularized value of the compressibility factor:
$$Z=\frac{\upsilon_{R}^{\left(w\right)}P_{c}}{k_{B}Tc}.$$

## Figures and Tables

**Figure 11.**
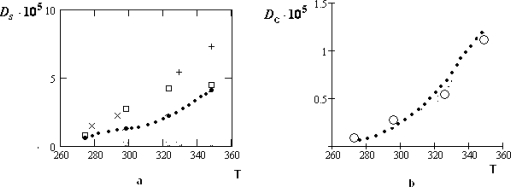
Temperature dependencies (• • •) of the self-diffusion coefficient *D_S_*, according to [36,38] (a) and its collective part *D_c_*, according to Equation (11); (b) for water molecules in the water solution of *LiCl* at *z_w_* = 6.05. Other symbols (× – [15], + – [16], – [36]) in (a) correspond to the experimental data for pure water.

**Figure 12.**
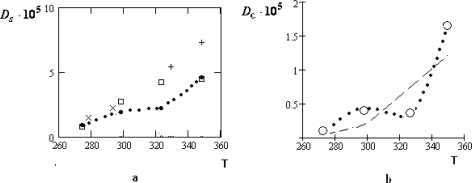
Temperature dependencies (• • •) of the self-diffusion coefficient *D_S_*, according to [36] (a) and its collective part *D_c_*, according to Equation (11); (b) for water molecules in the water solution of *NaCl* at *z_w_* = 6.05. All other designations have the same sense as in Figure 11.

**Figure 13.**
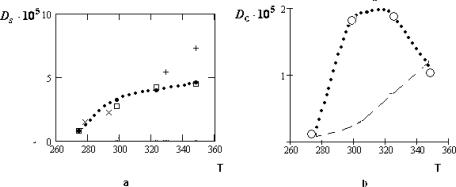
Temperature dependencies (• • •) of the self-diffusion coefficient *D_S_*, according to [36] (a) and its collective part *D_c_*, according to Equation (11); (b) for water molecules in the water solution of *KCl* at *z_w_* =6.05. All other designations have the same sense as in Figure 12.

**Table 1.** The values of *D_s_*, *D_c_* and $\tau_{0}^{\left(w\right)}$ for the water- *NaCl* solutions.

Solution	$r_{c}^{+}$, Ǻ	*z_w_*	*D_s_* ·10^5^, *cm*^2^/*s*	*D_c_* ·10^5^, *cm*^2^/*s*	$\tau_{0}^{\left(w\right)}\cdot 10^{12}s$
*W* – *NaCl*	0.98	27.8	2	0.45	0.7
The same	The same	13.9	1.75	0.43	0.9

**Table 2.** The values of $r_{c}^{+}$*D_s_* and *τ_0_* for the water solutions of *NaCl* and *KCl*.

Solution	$r_{c}^{+}$, Ǻ	*T*	*D_s_* ·10^5^, *cm*^2^/*s*	$D_{s}^{\left(w\right)}\cdot 10^{5}$, *cm*^2^/*s*	$\tau_{0}^{\left(h\right)}\cdot 10^{11}s$	$\tau_{0}^{\left(w\right)}\cdot 10^{11}s$
*W* – *NaCl*	0.98	348	4.6	7.3 [16]; 4.6 [36]	1	-
		323	2.7	4.2 [36]	1.2	0.02
		298	1.9	2.7 [36]	1.6	0.05
		274	0.9	0.8 [36]	1.8	0.2
*W* – *KCl*	1.33	348	4.3	7.3 [16]; 4.6 [36]	0.6	-
		323	3.4	4.2 [36]	0.7	0.02
		298	2.4	2.7 [36]	0.8	0.05
		274	1	0.8 [36]	0.9	0.2
